# Vie de couple et prise en charge de l'infection à VIH au service de pneumologie du CHU de Cocody, Abidjan, Côte d'Ivoire

**DOI:** 10.48327/MTSIBULLETIN.2021.123

**Published:** 2021-08-20

**Authors:** A. Koné, M.-O. Koffi, A. Djêgbéton, B.J.M. Ahui, V.C. Brou-Godé, A. N'gom, K. Horo, B.A. Kouassi, N.B. Koffi, E. Aka-Danguy

**Affiliations:** Les auteurs ne déclarent aucun conflit d'intérêt.

**Keywords:** VIH, Couple, Prévention, Stigmatisation, Discrimination, Anxiété, Dépression, Cocody, Abidjan, Côte d'Ivoire, Afrique subsaharienne, HIV, Couple, Prevention, Stigma, Discrimination, Anxiety, Depression, Cocody, Abidjan, Côte d'Ivoire, Sub-Saharan Africa

## Abstract

**Introduction:**

La vie de couple est un lieu de soutien émotionnel. Elle peut permettre d'optimiser la prise en charge des patients infectés par le VIH sous traitement antirétroviral.

L'objectif de notre étude était d'analyser l'impact de la vie de couple sur le suivi thérapeutique des patients vivant avec le VIH (PVVIH).

**Méthodologie:**

Nous avons réalisé une étude mono centrique, prospective, descriptive et analytique dans l'unité de prise en charge des PVVIH du service de pneumologie du CHU de Cocody à Abidjan. L'enquête s'est déroulée du 1^er^ septembre 2015 au 31 mars 2016.

**Résultats:**

Nous avons inclus 411 patients. Le sex-ratio était de 0, 51. La moyenne d’âge était de 43, 2 ans avec des extrêmes de 19 et 69 ans. Les patients vivaient en couple dans 59,1% des cas. La vie de couple était associée à l'immunodépression sévère à 12 mois dans 23,3% (42/180) des cas [p = 0, 043 OR = 1, 735 (0, 964 – 3, 121)], l'apparition de nouvelles affections opportunistes entre le 6^e^ et le 12^e^ mois de traitement dans 5,6% (13/232) des cas [p = 0, 006; OR = 9, 438 (1, 222 – 72, 890)], le partage de l'information avec le partenaire avant le début du traitement dans 92,4% (208/225) des cas [p = 0, 035; OR = 1, 976 (1, 005 - 3, 886)] et l'existence de rapports sexuels depuis la découverte de la maladie dans 92,6% (225/243) des cas [p<0, 001; OR = 14, 423 (8, 174 - 25, 448)]. Les rapports sexuels étaient moins protégés chez les personnes vivant en couple avec 65,9% (149/226) versus 78% (64/82) chez les autres [p = 0, 027; OR = 0, 544 (0, 301 - 0, 923)]. La perte du partenaire sexuel à l'annonce de la maladie était observée quelle que soit la situation matrimoniale (p = 0, 203).

**Conclusion:**

La vie de couple influence négativement la prise en charge de l'infection à VIH. Il apparaît nécessaire de mettre en place des programmes de conseil et dépistage s'adressant au couple.

## Abstract (en)

**Introduction:**

The life of a couple is a place of emotional support. It can allow the management of patients infected with HIV on antiretroviral therapy to be optimized.

The objective of our study was to analyze the impact of married life on the therapeutic follow-up of patients living with HIV.

**Methodology:**

We carried out a mono-centric, prospective, descriptive and analytical study in the care unit of patients living with HIV of the pneumology service of the Cocody Teaching Hospital in Abidjan. The investigation took place from September 1, 2015 to March 31, 2016.

**Results:**

We included 411 patients. The sex ratio was 0.51. The average age was 43.2 years with extremes of 19 and 69 years. The patients lived as a couple in 59.1% of cases. Couple life was associated with severe immunosuppression at 12 months in 23.3% (42/180) of cases [p = 0.043 OR = 1.735 (0.964 - 3.121)], the appearance of new opportunistic conditions between the 6th and the 12th month of treatment in 5.6% (13/232) of cases [p = 0.006; OR = 9.438 (1.222 - 72.890)], information sharing with the partner before the start of treatment in 92.4% (208/225) of cases [p = 0.035; OR = 1.976 (1.005-3886)] and the existence of sexual intercourse since the discovery of the disease in 92.6% (225/243) of cases [p < 0.001; OR = 14.423 (8.174 - 25.448)]. Sexual relationships were less protected among people living in a couple 65.9% (149/226) versus 78% (64/82) among others [p = 0.027; OR = 0.544 (0.301 - 0.923)]. The loss of the sexual partner at the onset of the disease was observed regardless of marital status (p = 0.203).

**Conclusion:**

The life of a couple negatively influences the management of HIV infection. It appears necessary to set up counseling and testing programs for couples.

## Introduction

La Côte d'Ivoire compte en 2019, 430 mille personnes vivant avec le VIH dont 63% sous trithérapie antirétrovirale [7]. C'est à présent une affection chronique dont la prise en charge est facilitée par l'existence de trithérapies efficaces entièrement gratuites en Côte d'Ivoire depuis 2008 [4]. Cette gratuité a permis une réduction de la létalité et une baisse de la prévalence de l'infection passée de 3,7% en 2011-2012 à 2,4% en 2019 [2, 7].

Dans l'optique de rechercher d'autres facteurs qui pourraient permettre de maitriser à long terme ce fléau, des études ont montré une relation entre le statut social et l’évolution de la maladie [3, 8]. Par exemple, il a été démontré que le maintien ou la construction d'une vie de couple constituait une ressource identitaire et un soutien fort face à l'infection à VIH. De même l'existence d'une relation stable a été associée à une évolution favorable de la maladie [3, 10].

Ainsi, au vu des difficultés dans l’épanouissement social en particulier sentimental des personnes vivant avec le VIH et des avantages liés à la vie de couple, nous nous sommes proposés d'analyser l'impact de la vie de couple sur le suivi thérapeutique des patients vivant avec le VIH.

## Méthodologie et population étudiée

Notre étude monocentrique, prospective, descriptive et analytique s'est déroulée du 1^er^ septembre 2015 au 31 mars 2016 dans le service de pneumologie du CHU de Cocody au sein de l'Unité de prise en charge des personnes vivant avec le VIH (PVVIH).

Nous avons inclus tous les patients âgés de 15 ans au moins, suivis dans l'unité de prise en charge des PVVIH et sous antirétroviraux (ARV) depuis au moins 12 mois et pour qui un consentement éclairé, écrit et signé a été obtenu.

Nous n'avons pas inclus les patients transférés d'un autre centre, ceux dont les dossiers n'ont pu être retrouvés ou ceux ayant refusé de donner leur consentement.

La vie de couple était définie comme étant le fait d’être marié ou de vivre en union libre sous le même toit sans préjuger de la durée.

L'anxiété et la dépression ont été mesurées avec le questionnaire de l’échelle *Hospital Anxiety and Depression scale* (HAD) de Zigmund et Snaith [11] validé chez les patients infectés par le VIH. L’échelle HAD vise à évaluer le niveau de symptomatologie dépressive et anxieuse. Elle comprend 14 critères, dont 7 évaluant l'anxiété et 7 la dépression. Pour chacun d'eux, 4 réponses étaient cotées, de sévérité croissante de 0 à 3 ou décroissante de 3 à 0. L'anxiété était dite certaine lorsque le score HAD anxiété était ≥ 10. La dépression était considérée certaine pour un score HAD dépression ≥ 10.

L'immunodépression sévère était retenue lorsque le taux de lymphocytes T CD4 était < 200/ml.

La prise en charge correcte de l'infection est définie par:

- la surveillance clinique régulière, l'initiation du traitement à 2 semaines, puis tous les mois pendant 3 mois et enfin tous les 3 à 6 mois;
- la surveillance biologique tous les 6 mois.
Les paramètres de l’étude répertoriés dans une fiche d'enquête étaient :
- les variables sociodémographiques;
- les aspects du diagnostic de l'infection VIH;
- les aspects cliniques et paracliniques du suivi sous ARV (l'examen clinique, le taux de lymphocytes CD4, la charge virale, le bilan biologique standard et la radiographie du thorax);
- le partage de l'information, les comportements sexuels;
- les connaissances sur le VIH;
- l'anxiété-dépression.

Nous avons abordé les patients le jour de la réalisation de leur bilan de suivi ou de la dispensation des ARV. Après leur avoir expliqué l'intérêt de l’étude, le consentement était requis pour l'inclusion dans l’étude si le patient remplissait les critères de sélection. La fiche d'enquête était renseignée par le patient en présence de l'enquêteur. Lorsqu'il s'agissait d'un mineur, le consentement était recueilli auprès du représentant légal et quand le patient ne parlait pas français, l'enquêteur se faisait aider par un proche de celui-ci qui servait de traducteur. L'enquête s'est déroulée du 1er septembre 2015 au 31 mars 2016.

Nous avons utilisé les proportions et les moyennes pour décrire les résultats généraux. Les comparaisons des proportions ont été faites avec le test de chi^2^ et de Fisher. Le seuil de p < 0, 05 a été considéré significatif. Les données ont été analysées avec le logiciel SPSS 20.0.

## Résultats

### Résultats généraux

Au cours de cette enquête, 411 PVVIH ont été inclus. Le sex-ratio était de 0, 51. La moyenne d’âge était de 43, 2 ans avec des extrêmes de 19 et 69 ans. Les patients vivaient en couple dans 59,1% des cas (243/411). Il s'agissait de couples hétérosexuels dans tous les cas. La durée moyenne de suivi de l'infection VIH au moment de l’étude était de 54, 73 mois avec des extrêmes de 17 et 175 mois. Au moment du diagnostic de l'infection VIH, 34,5% (142/411) avaient des infections opportunistes (tuberculose, mycoses, pneumocystose) et 63,5% (261/411) étaient au stade d'immunodépression sévère avec un taux de lymphocytes T CD4 < 200/ml. On notait une interruption du traitement antirétroviral dans 4,3% (17/391) des cas la première année de suivi. Les raisons de cette interruption étaient les croyances religieuses 5,9% (1/17), les ruptures de stocks 17,6% (3/17), les effets secondaires des médicaments 35,3% (6/17) et les difficultés financières liées au déplacement 41,2% (7/17). En effet, 22% des patients vivaient en zone rurale (90/411).

Les modes de transmission de l'infection à VIH étaient connus dans 94% des cas (386/411) et 92,5% (380/411) des patients ont affirmé avoir partagé leur statut sérologique avec une tierce personne. Les personnes les plus informées étaient la fratrie 28% (199/710), suivies du conjoint 26% (184/710), puis des parents 15% (104/710) et amis 11% (80/710). Ils ont pensé que la divulgation de leur séropositivité pouvait faire perdre l'estime des autres à leur égard dans 22% des cas (90/411) tandis que 7,5% (31/411) ont affirmé avoir été victimes d'injustice du fait de l'infection par le VIH. Par ailleurs, après l'annonce de la maladie, 4,6% des patients (19/411) ont été rejetés par leur partenaire. La dépression certaine était de 0,2% (1/411) de l'ensemble de l'effectif et l'anxiété certaine de 2,9% (12/411).Depuis la découverte de l'infection, 69,2% (213/308) des patients ont eu des rapports sexuels avec leur partenaire. Ceux-ci étaient protégés dans 69,2% des cas (213/308).

### Résultats analytiques

#### Vie de couple et aspect clinique

Au moment du diagnostic de l'infection à VIH, il n'a pas été possible de montrer une différence significative de la présence d'une immunodépression sévère entre les patients vivant en couple 63,4% (154/243) et ceux ne vivant pas en couple 63,7% (107/168) [p = 0, 516 OR = 0, 986 (0, 656 - 1, 484)]. Cependant la proportion d'immunodépression sévère à 12 mois était de 23,3% (42/180) en cas de vie de couple contre 14,9% (20/134) dans le cas contraire [p = 0, 043 OR = 1, 735 (0, 964 – 3, 121)]. De plus, on notait l'apparition de nouvelles affections opportunistes entre le 6^e^ et le 12^e^ mois de traitement chez les patients vivant en couple dans 5,6% (13/232) des cas contre 0,6% (1/160) [p = 0, 006; OR = 9, 438 (1, 222 - 72, 890)]. Nous n'avons pas trouvé de différence significative d'une interruption du traitement la première année entre les patients en couple 4,3% (10/232) et ceux qui ne vivaient pas en couple 3,8% (6/159) [p = 0, 505 OR = 1, 149 (0, 409 - 3, 227)].

#### Vie de couple et comportements

Le partage de l'information avec le partenaire avant le début du traitement était plus fréquent chez les patients vivant en couple 92,4% (208/225) versus 86,1% (130/151) au sein des sujets non en couple [p = 0, 035; OR = 1, 976 (1, 005 - 3, 886)]. Après l'annonce de l'infection, l'existence de nouveaux partenaires sexuels était similaire quel que soit le statut matrimonial 34,4% (83/241) versus 38,9% (65/167) [p = 0, 206; OR = 0, 824 (0, 548 - 1, 241)]. La proportion de patients ayant eu des rapports sexuels depuis la découverte de la maladie était plus élevée chez les patients en couple 92,6% (225/243) versus 38,7% (65/168), chez ceux sans vie de couple [p < 0, 001; OR = 14, 423 (8, 174 – 25, 448)]. Les rapports sexuels étaient protégés moins souvent chez les personnes vivant en couple 65,9% (149/226) versus 78% (64/82) chez les autres [p = 0, 027; OR = 0, 544 (0, 301 - 0, 923)] (Tableau I).

**Tableau I:** Vie de couple et comportement Couple life and behaviour

	Vie de couple		P	OR, IC_95%_
	Oui	Non		
**Partage de l'information**	93,8% (228/243)	90,5% (152/168)	0, 141	1, 600 (0, 768- 3, 332)
**Nouveaux partenaires**	34,4% (83/241)	38,9% (65/167)	0, 206	0, 824 (0, 548 – 1, 241)
**Rapports sexuels depuis la découverte de l'infection**	92,6% (225/243)	38,7% (65/168)	<0, 001	14, 423 (8, 174 – 25, 448)
**Utilisation de préservatifs**	65,9% (149/226)	78% (64/82)	0, 027	0, 544 (0, 301 – 0, 983)

#### Vie de couple et conséquences du partage

Le départ du partenaire sexuel à l'annonce de la maladie était observé, quelle que soit la situation matrimoniale (p = 0, 203). La dépression certaine n’était pas associée à la vie de couple (p = 0, 591) de même que l'anxiété certaine (p = 0, 107) (Tableau II).

**Tableau II:** Vie de couple et conséquences du partage Couple life and consequence of serological status sharing

	Vie de couple		P	OR, IC_95%_
	Oui n = 243	Non n = 168		
**Perte du partenaire sexuel**	9 3,7%	10 6%	0, 203	0, 608 (0, 241 – 1, 529)
**Dépression certaine**	1 0,4%	0 0%	0, 591	1, 694 (1, 563 – 1, 837)
**Dépression probable**	4 1,6%	7 4,2%	0, 107	0, 385 (0, 111 – 1, 336)

## Discussion

Au cours de cette étude, on décrit une population jeune, majoritairement féminine, dont plus de la moitié vivait en couple. Les personnes vivant en couple avaient plus fréquemment une immunodépression sévère après 12 mois de traitement et étaient plus souvent sujettes à de nouvelles affections opportunistes entre 6 et 12 mois de traitement. Le partage de la séropositivité au sein du couple se faisait avant le début du traitement. Les patients vivant en couple avaient plus fréquemment des rapports sexuels depuis la découverte de la maladie et ces rapports étaient moins souvent protégés. Il n'y avait pas de conséquences psychosociales liées au mode de vie en couple ou non.

La proportion élevée de patients en couple était similaire à celle retrouvée en Guadeloupe avec 30,4% de séropositifs en couple [5].

L'immunodépression fréquente après la première année de traitement et l'existence de nouvelles affections opportunistes chez ces patients s'expliqueraient par la mauvaise observance thérapeutique la première année du diagnostic de l'infection. En effet, malgré la prise en charge gratuite de l'infection, il existait des coûts liés au déplacement ne leur permettant pas de s'approvisionner régulièrement en médicaments et ces coûts seraient plus sensibles pour les personnes vivant en couple.

La faible utilisation du préservatif est plus souvent retrouvée dans les couples stables qu'avec les partenaires occasionnels [5, 6]. En effet, selon une étude réalisée à Abidjan [1], 64% des couples n'utilisaient jamais de préservatifs. Ce refus de l'utiliser s'expliquait par la réduction du plaisir, le désir de maternité, les raisons religieuses et surtout la peur de le proposer au partenaire. Il est aussi associé à la preuve d'une infidélité du partenaire qui le propose. La découverte de l'infection au sein du couple ne constituait pas un frein à la poursuite de la vie sexuelle. Selon une étude réalisée en Suisse, les patients infectés par le VIH avaient continué les rapports sexuels au cours des 12 derniers mois et ces rapports étaient plus fréquents avec des partenaires stables qu'occasionnels [10].

L'annonce de la séropositivité reste un élément de stress, du fait du risque de stigmatisation, de la peur du rejet et surtout de la peur de contaminer son conjoint. Quand le partage de l'information est fait au sein du couple, celui-ci peut être mis à rude épreuve aboutissant parfois à des ruptures [9]. Dans notre contexte, l'annonce est motivée par la confiance et le soutien aussi bien psychologique que moral que les proches apportent aux patients. Selon des études, 16,7% à 86% des personnes partagent leur statut en Afrique subsaharienne [1]. Le partage se fait beaucoup plus avec l'entourage familial qu'avec le conjoint [5]. Dans l’étude ANRS-VESPA, le partage de l'information ne se faisait pas toujours [5, 6]. L'infection à VIH reste encore associée à une représentation négative malgré les campagnes de déstigmatisation. Ainsi la dégradation de l'image de soi, la peur du rejet, le déni de la maladie et la remise en cause du droit à la maternité ou à la paternité sont autant de raisons qui empêchent le partage de l'information au sein du couple. Dans notre série, le partage de l'information au sein du couple n'entraînait pas de conséquences psychosociales.

Notre étude présente des limites, car elle ne présente pas de suivi des patients sur une longue période. Il existe peu d’étude réalisée sur le suivi au long cours des patients, la plupart étant des études transversales. De plus, l’étude a été réalisée dans un seul centre de la zone urbaine d'Abidjan, capitale économique de la Côte d'Ivoire mais près du tiers de notre population d’étude vivait en zone rurale. Il serait difficile d'extrapoler nos résultats à l’échelle nationale.

En outre, nous avons été confrontés à d’énormes difficultés devant le remplissage du questionnaire HAD. La Côte d'Ivoire, pays en voie de développement, présente un fort taux d'analphabétisme. Pour pallier ce fait, l'enquêteur a dû traduire parfois certaines parties du questionnaire. La question de la sexualité était un sujet tabou pour bon nombre de nos patients. De plus la notion de couple en Afrique inclut parfois la notion de polygamie qui n'a pas pu être prise en compte dans cette étude.

## Conclusion

Cette étude a permis de montrer que la vie de couple n'améliore pas l’évolution clinique des patients. Elle a été associée à une apparition d'infections opportunistes et une immunodépression sévère après 12 mois de traitement ARV. Par ailleurs elle ne semble pas influencer l'incidence des conséquences psychosociales: anxiété et dépression sont aussi fréquentes dans les 2 groupes. Face à ce constat, il apparaît opportun de mettre en place un programme de conseil et de dépistage qui s'adresse aux couples afin de permettre une bonne adhésion à la prise en charge de l'infection à VIH.

## Conflits D'intérêts

Les auteurs ne déclarent aucun conflit d'intérêt.
